# Countries’ progress for women’s and children’s health in the Millennium Development Goal era: the Countdown to 2015 experience

**DOI:** 10.1186/s12889-016-3398-x

**Published:** 2016-09-12

**Authors:** Peter Berman, Jennifer Requejo, Zulfiqar A. Bhutta, Neha S. Singh, Helen Owen, Joy E. Lawn

**Affiliations:** 1 Harvard T. H. Chan School of Public Health, 677 Huntington Ave, Boston, MA 02115 USA; 2 Johns Hopkins Bloomberg School of Hygiene and Public Health, 615 N Wolfe St, Baltimore, MD 21205 USA; 3 Aga Khan University, Stadium Rd, Karachi, 74800 Pakistan; 4 Centre for Global Child Health, The Hospital for Sick Children, 686 Bay Street, Toronto, Ontario M5G 0A4 Canada; 5 Dalla Lana School of Public Health, University of Toronto, Toronto, Canada; 6 Centre for Maternal, Adolescent, Reproductive and Child Health, London School of Hygiene & Tropical Medicine, London, WC1E 7HT UK

Countdown to 2015 for Maternal, Newborn and Child Survival (Countdown) began in 2003, monitoring and analysing country progress towards achieving Millennium Development Goals (MDGs) 4 (reduce child mortality) and 5 (improve maternal health), responding to *The Lancet* Child Survival Series [1]. For 12 years Countdown synthesised data on coverage and its key determinants across the continuum of care for women’s and children’s health, and regularly disseminated country profiles, reports, and publications in high impact journals [2]. Understanding global level progress to the MDGs was more recently complemented by a series of country case studies (Afghanistan, Bangladesh, China, Ethiopia, Kenya, Malawi, Niger, Pakistan, Peru, and Tanzania), many are accompanied by national review meetings to inform policy formulation and programme implementation [3–8]. This supplement collates learning from these analyses.

Through global and country level tracking efforts, Countdown provided an objective assessment of progress towards MDGs 4 and 5, advancing global accountability for women’s and children’s health, feeding into the work of the Independent Expert Review Group on Information and Accountability for Women’s and Children’s Health (iERG) for the UN Secretary General [9]. Countdown’s “suprainstitutional” structure, including academics, governments, international agencies, professional associations, donors, and members of civil society, helped to ensure independence and credibility with wide uptake.

Countdown to 2015 closed with its final report [10], and re-opened as Countdown to 2030 in the spring of 2016. The process of re-designing the initiative to be responsive to the Sustainable Development Goal Framework and the new Global Strategy for Women’s, Children’s and Adolescents’ health involved reflecting on Countdown’s country orientation. The core of Countdown reporting since its inception has been the two-page country profiles for each of the 75 priority countries where more than 95 % of all maternal, newborn and child deaths occur. The profiles present demographic indicators; nutritional status and mortality statistics; coverage levels, trends and equity analyses for evidence-based reproductive, maternal, newborn and child health interventions, plus policy, health systems, and financial indicators. The profiles are a visual tool to help policy makers and development partners to identify gaps to address and work to maintain progress.

In addition to carrying out analyses for the country profiles and summary reports, Countdown’s four technical working groups (coverage, equity, health systems and policies, financing) worked together on cross-cutting research with the aim of using the global databases to systematically examine linkages between intervention coverage, its determinants and improvements in survival. Countdown experts identified limitations inherent in various surveys and databases, making causal inferences at the aggregate level very challenging. For example, the Countdown coverage database maintained by Johns Hopkins University in collaboration with UNICEF depends primarily on household survey data, principally the Demographic and Health Surveys and the Multiple Indicator Cluster Surveys. Not all Countdown countries have new household survey data in a given year, and the indicators reported by Countdown reflect information on intervention coverage over different recall periods (e.g. the recall period for skilled attendance at birth is often 3 years prior to the survey and exclusive breastfeeding is the 24 hours before). The Countdown health policy database maintained by the World Health Organisation (WHO), although it represents a major step forward in being able to track policy indicators related to Reproductive, Maternal, Newborn and Child health (RMNCH), does not include information on exactly when a policy was adopted and its roll out via programmes in each country, making it challenging to link policy adoption to trends in intervention coverage or financial flows to RMNCH. Countdown’s database on financing focuses on external resource flows, and there has been no comparable data on domestic funding linked to MDGs 4 and 5 for a large group of countries. An effort by some Countdown researchers to explain the relative contribution of different factors to MDG progress through an aggregate cross-sectional and time series analysis of all 75 Countdown countries proved to have little explanatory power, in part due to these limitations.

Countdown also recognised the importance of contextual factors (e.g., economic and social development progress, women’s social status or conversely, conflict, emergencies) in influencing progress. Variation across countries meant that ecological analyses could only go so far in explaining how change was achieved or not at national and subnational levels.

In view of these issues, Countdown embarked upon a set of country case studies that would enable an assessment of why and how countries progressed towards improving women’s and children’s health during the MDG-era, and what factors might explain the specific patterns of progress in individual countries. The first case study was undertaken in Niger and involved a close collaboration between Johns Hopkins University, the National Statistics Office, and the UNICEF country office. Its publication in 2012 in *The Lancet* accompanied by a comment penned by the Minister of Health of Niger generated an enthusiastic response from countries for a similar analysis [6]. In response, Countdown developed a case study model adapted from this approach and selected nine additional countries for analysis. The full portfolio includes 10 countries at varying stages of progress towards MDGs 4 and 5. These studies involved a partnership between academic institutions well versed in Countdown’s global work and country-based teams consisting of members not directly involved in programme implementation or evaluation to safeguard the independence of the analysis. Each case study collaborative took advantage of country-specific opportunities for learning and faced unique challenges related to the different circumstances in the country (e.g. data availability, team capacity/attrition, and communication between agencies).

This journal supplement describes key lessons from the Countdown case study portfolio. It includes a combination of a summary paper [11] and two methodological papers synthesising findings across the case studies [12] and showcasing novel methods developed for the case studies [13], and three country specific papers that use subnational data to explore specific themes across the continuum of care (Table 1). The summary paper, by Moucheraud et al., distils key factors that detracted from and facilitated progress across the RMNCH continuum of care in the case study countries [11]. The second paper by Singh et al. showcases results from a set of novel tools used by the case study teams to analyse country policies and health systems factors associated with change [13]. The second methods paper by Mann et al. provides a cross-country comparison of how financial flows from domestic and external sources influence RMNCH outcomes [12]. The Tanzania case study subnational analysis by Armstrong et al. focuses on variations in progress at the regional level in increasing coverage and improving health system readiness to deliver essential interventions around the time of birth [14]. The Peru analysis by Huicho et al. examines national and district level trends in the neonatal mortality rate by wealth quintile and by urban/rural residence, and factors underlying these trends [15]. Finally a paper from Afghanistan by Akseer et al. uses the Lives Saved Tool to estimate impact based on RMNCH services coverage in eight regions, showing progress despite major contextual challenges [16].

**Table 1:** Countdown to 2015: country case studies supplement objectives and overview

**Overall supplement objectives** 1) To use evidence from the portfolio of in-depth country case studies to describe lessons learned, focusing on how progress was achieved. 2) To showcase results from Countdown country case studies that used a set of novel health system and policy (HSP) tools designed to systematically analyse HSP factors that have contributed to change, or lack thereof, in reproductive, maternal, newborn and child health (RMNCH) in a country, how these changes occurred, and to enable cross-country comparisons. 3) To provide a cross-country comparison on the health financing component of the case studies, analysing how health financing may be related to RMNCH outcomes. 4) To present evidence on regional variation across health systems building blocks for care at birth outcomes in Tanzania, to test associations between health system inputs and outcomes, and to present each region’s implementation readiness according to key inputs and performance. 5) To describe the variations in neonatal mortality over time in Peru, to explore disaggregated trends by wealth quintiles and by urban/rural residence over time, to assess completeness of registered deaths, and to explore possible factors driving the progress achieved 6) To explore coverage and socioeconomic inequalities in key life-saving RMNCH interventions at the national level and by region in Afghanistan. **Overview of papers in the supplement** ***Paper 1***: *Multi-country analyses*: Mixed method (quantitative and content analyses) evidence from the portfolio of country case studies to describe lessons learned, focusing on how progress was achieved across the RMNCH continuum of care [11]. ***Paper 2***: *Health policies and systems:* Highlights the methods and key findings from the health systems and policies tools developed for the purpose of the case studies to understand key factors driving progress across the RMNCH continuum of care in case study countries [13]. ***Paper 3***: *Financing:* Draws on lessons learned and overall themes from the Countdown case studies on how health financing influences RMNCH outcomes and the achievements of MDG 4 and 5 [12]. ***Paper 4***: *Tanzania:* Evaluates subnational (at regional level) variations for care at birth outcomes, i.e. rural women giving birth in a facility and by Caesarean-section, and associations with inputs according to the health systems building blocks [14]. ***Paper 5***: *Peru:* Describes time trends in NMR at national and district level in Peru, by wealth quintiles and by urban/rural residence and explore underlying factors [15]. ***Paper 6***: *Afghanistan:* A ssesses levels of coverage, and the absolute and relative socioeconomic inequalities in 11 essential RMNCH interventions, including measures of composite coverage, at the national level and for the eight geographic regions of Afghanistan. To quantify the number of child deaths averted through scale up of effective community-based interventions across socioeconomic groups based on the Lives Saved Tool (LiST) [16].	

Some learnings from the Countdown case studies evidence and experience include:

- A *common framework* is helpful to structure the evaluation and especially to consider links between impact with the various domains of Countdown technical work (coverage, equity, finance and health policy and systems) (Paper 1 Fig. 1) [11].
- Comparability across countries and sub-nationally is easier to achieve for *quantitative assessments* of coverage and equity, and the Countdown has particularly advanced the use of equity analyses to better identify which regions or income groups or other populations are being left behind.
- To date, less attention has been paid to comparability for the *evaluation of inputs and process*, such as finances [12] and health policy and systems [13]. Countdown has made some progress in more standard tools and approaches but more work is needed for both international comparisons and subnational analyses.
- The Countdown case studies mostly focused more on child health interventions than on reproductive, maternal, and newborn health, perhaps due to more time series data availability during the study period. More effort is needed to examine progress across the *continuum of care* including comparisons of rates of progress between different outcomes and nationally/subnationally, and neglected age groups such as adolescents and stillbirths.
- *Many factors contribute to progress, or lack of progress*, in individual countries. We found that no one country or group of countries should be seen as a “success” or “failure”. The value of the case studies is in understanding variations and learning from successes and setbacks, not causal hypothesis testing [11]. Richer and more standardised assessments would be valuable to consider the why and how of change, including economic, political, social and governance factors.
- Progress was most evident for *relatively simpler interventions delivered at the community-primary care interface* and lagging or uneven for those typically found in health facilities or requiring 24h services. This highlights the importance of more systematic health system strengthening, especially with an expanded SDG agenda.
- *Engagement in national data analysis and accountability* requires significant time commitment in multi-stakeholder processes and leadership from committed academics and researchers skills in monitoring, evaluation and policy change. Support from technical experts and organisations outside of study countries is valuable to take advantage of methods and analytical tools from elsewhere. To maximise impact and sustainability, more effort is required for strengthening capacity in national institutions, and to integrate findings into country planning and review cycles. These efforts go beyond those typical of research studies [17].
- Dissemination and linking to accountability is not a random process and requires specific plans and local leadership, with civil society voices involved.

Building on the case study experience, Countdown to 2030 includes will be more focused on in-country work and local capacity for monitoring and evaluation. Analyses will include countries making progress as well as those where gains are slower. At global level, Countdown will expand to hitherto ignored groups such as adolescents, marginalised populations, and those affected by conflict. If Countdown could finish at the end of the SDG-era in 2030 with an empowered community of scientists and advocates in the very countries with the highest burden of maternal and child morbidity and mortality, this would be well worth the investment.
